# Alzheimer’s disease and antibody-mediated immune responses to infectious diseases agents: a mendelian randomization study

**DOI:** 10.1186/s41065-024-00358-4

**Published:** 2025-01-04

**Authors:** Jiayuan Zhang, Mingming Wang, Dong Wang, Linwen Deng, Yao Peng

**Affiliations:** 1 Sichuan Police College, No. 186 Longtouguan Road, Jiangyang District, Luzhou, Sichuan 646000 China; 2 Department of Gynecology, Hospital of Chengdu University of Traditional Chinese Medicine, No. 37 Shi er’qiao Road, Jinniu District, Chengdu, Sichuan China; 3 The Affiliated Traditional Chinese Medicine Hospital, Southwest Medical University, No. 182 Chunhui Road, Longmatan District, Luzhou, Sichuan 646000 China; 4 Nanjing University of Chinese Medicine, No. 138 Xianlin Road, Qixia District, Nanjing, Jiangsu 210023 China

**Keywords:** Antibodies, Alzheimer’s disease, Causality, GWAS, Mendelian randomization

## Abstract

**Background:**

Alzheimer’s disease (AD) is a prevalent neurodegenerative disorder, with antibody-mediated immune responses to infectious diseases agents potentially playing a decisive role in its pathophysiological process. However, the causal relationship between antibodies and AD remains unclear.

**Methods:**

A two-sample Mendelian randomization (MR) analysis was conducted to investigate the causal link between antibody-mediated immune responses to infectious diseases agents and the risk of AD. Genetic variations associated with these antibodies obtained from UK Biobank, and data on AD were obtained from the Finnish databases, utilizing its extensive repository of genome-wide association studies (GWAS) for a comprehensive analysis. The MR analysis employed the inverse variance-weighted, MR-Egger, and weighted median methods. Sensitivity analysis was also performed using MR-Egger regression, MR-pleiotropy residual sum, and outlier tests.

**Results:**

Two causal associations were identified between antibody-mediated immune responses to infectious diseases agents and AD. Varicella zoster virus glycoproteins E and I antibody suggest a protective association with AD. Conversely, higher levels of Epstein-Barr virus EBNA-1 antibody appear to be associated with an increased risk of AD.

**Conclusion:**

Our MR analysis has revealed a causal relationship between antibody-mediated immune responses to specific infectious disease agents and AD. These findings provide valuable insights into the pathophysiological mechanisms underlying AD.

**Supplementary Information:**

The online version contains supplementary material available at 10.1186/s41065-024-00358-4.

## Introduction

Alzheimer’s disease (AD) is the most prevalent neurodegenerative disorder and the leading cause of dementia among the elderly. Clinical manifestations of AD include memory impairment, difficulties in abstract thinking and computational abilities, and alterations in personality and behavior [1, 2]. The characteristic pathological changes in AD involve the excessive deposition of Aβ plaques, hyperphosphorylated tau protein forming neurofibrillary tangles, and neuroinflammatory responses due to overactivated microglia [3, 4]. In 2019, the International Alzheimer’s Association estimated that over 50 million people worldwide suffer from AD, with projections indicating a doubling of cases by 2050, significantly escalating the socio-economic burden [5]. Despite being identified over a century ago, the precise pathogenesis of AD remains incompletely understood.

Traditionally, the brain was considered a sterile environment due to the presence of the blood-brain barrier. However, recent studies have revealed the presence of bacterial communities in post-mortem human brain tissues, suggesting that microorganisms may directly affect the brain [6]. There is substantial evidence linking AD with various pathogens, including human herpesviruses, Chlamydia pneumoniae, Helicobacter pylori, and several periodontal pathogens [7]. Emerging research indicates that β-amyloid protein may serve physiological roles, acting as a defense mechanism against potential AD-inducing infections by viruses, bacteria, and fungi. It functions as a natural antimicrobial peptide [8] and can directly kill pathogens [9]. Patients infected with herpes simplex virus have a twofold increased risk of developing dementia in old age compared to controls [10]. Active antiviral treatment in HSV-infected individuals can prevent AD onset up to 10 years later. Another study demonstrated that populations successfully eradicating **H. pylori** exhibited improved cognitive function after a 2-year follow-up [11]. Animal experiments also revealed increased astrocyte numbers in the brains of Hp-infected mice, indicative of neuroinflammation [12, 13]. Although multiple studies have established a connection between pathogen infection and AD, the specific causal relationship remains contentious. Therefore, understanding the potential causal relationship between pathogen infection and AD is crucial for the prevention and treatment of AD.

To address the inherent potential confounding factors in observational studies, MR provides an effective method. This approach introduces intermediate variables, namely instrumental variables (IVs), to analyze the causal relationship between exposure factors and outcomes. Utilizing publicly available genome-wide association studies GWAS data, our study aims to investigate the potential role of antibody-mediated immune responses to infectious agents in the pathogenesis of AD. We employed a two-sample MR analysis to assess this relationship. Additionally, we evaluated the association between genetic predisposition to AD and antibody-mediated immune responses to infectious agents, thereby gaining a clearer understanding of the interplay between AD and these immune responses.

## Method

### Study design

**Fig. 1:**
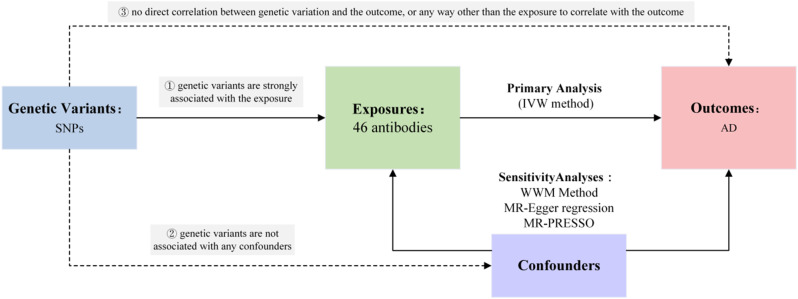
The mendelian randomization study design

In the current study, pooled data from the published genome-wide association studies (GWASs) of 46 antibodies and AD were utilized. Firstly, genetic variants associated with each antibody were selected to identify the causal relationship of each antibody with AD. Secondly, AD-associated genetic variants were employed to infer causality between AD and antibodies, respectively. The MR approach relied on three key assumptions: (1) Strong association of genetic variants used as instrumental variables with exposure; (2) No association of genetic variants with any confounders; (3) Absence of a direct correlation between genetic variation and the outcome, except through exposure [14]. As all data used were publicly available, no additional ethical approval was required. The specific study design is depicted in Fig. 1.

### GWAS sources

The GWAS statistics of AD were also derived from the FinnGen consortium R11, with 11,755 cases and 441,978 controls, which were defined as Alzheimer’s disease [15]. The exposure data source for this MR study is the UK Biobank, which recruited over half a million British adults between 2006 and 2010. Among these, a subsample of 9,724 participants provided serum samples for serological measurements of 20 different microorganisms. These samples were tested for total antibody levels against multiple antigens [16].

### The selection of IVs

In our Mendelian randomization analysis, we employed IVs as tools to discern the causal relationships between exposure factors and outcomes. IVs are typically genetic variants, with SNPs being the variant of choice for their commonality. To ensure an adequate number of SNPs for further MR analysis, a significance threshold of *P* < 5 × 10^− 6^ was selected when screening for SNPs related to each antibody and AD. a substantial clumping window over 10,000 kb, and minimal linkage disequilibrium (*r*^*2*^ < 0.001), as these measures ensure the SNPs’ independence [17]. Moreover, to affirm the robustness of the association between IVs and the exposure, we utilized the F statistic as a gauge of validity [18]. In this study, the inclusion criteria for SNPs demanded F values exceeding 10, thus minimizing the risk of weak instrument bias. Palindromic SNPs, specifically those with A/T or G/C alleles, were systematically identified and excluded to prevent the ambiguity they can cause regarding the effect allele in GWAS studies of both exposure and outcome. To address and rectify the presence of horizontal pleiotropy, we employed the Mendelian Randomization Pleiotropy RESidual Sum and Outlier (MR-PRESSO) tool for both global and outlier analyses, rooting out SNPs exhibiting such effects [19].

### Statistical analysis

To estimate the causal effect, we utilized the inverse variance weighted (IVW) method as our primary analytical approach. The IVW model is particularly potent for detecting causality within the framework of two-sample MR analysis [20]. We translated our findings into odds ratios (ORs) and their corresponding 95% confidence intervals (CIs). Heterogeneity within the IVW estimates was evaluated using Cochran’s Q test, where a p-value less than 0.05 signified heterogeneity. Nevertheless, heterogeneity does not inherently invalidate the IVW model. Given the diversity in causal estimates from different variants, the multiplicative random effects model was deemed preferable to the fixed effects model, and was therefore adopted for our main analysis.

Furthermore, we employed the MR-PRESSO method to identify and correct for outliers in the IVW linear regression [19]. The MR-Egger method, which accommodates non-zero intercepts, was applied to check for the presence of directional pleiotropy. We also conducted leave-one-out analyses to determine the influence of individual SNPs on our results. Using MR-PRESSO, we were vigilant in detecting and excluding outliers promptly. Following outlier removal, the MR analyses were revisited. All computations and statistical analyses were facilitated by the R software (version 4.3.2), utilizing the TwoSampleMR package. We applied a significance threshold of *P* < 0.001 to account for multiple testing, ensuring a stringent control of type I error given the 46 antibodies analyzed. This adjustment minimizes the likelihood of false-positive results and enhances the robustness of the causal inferences drawn. In cases where IVW analysis yielded a significant result (*P* < 0.001) and no evidence of horizontal pleiotropy or heterogeneity was found, the result could be considered positive, even if other methods did not yield a significant result, as long as the direction of the beta remained consistent.

## Results

### Causal impact of antibodies on AD

**Fig. 2:**
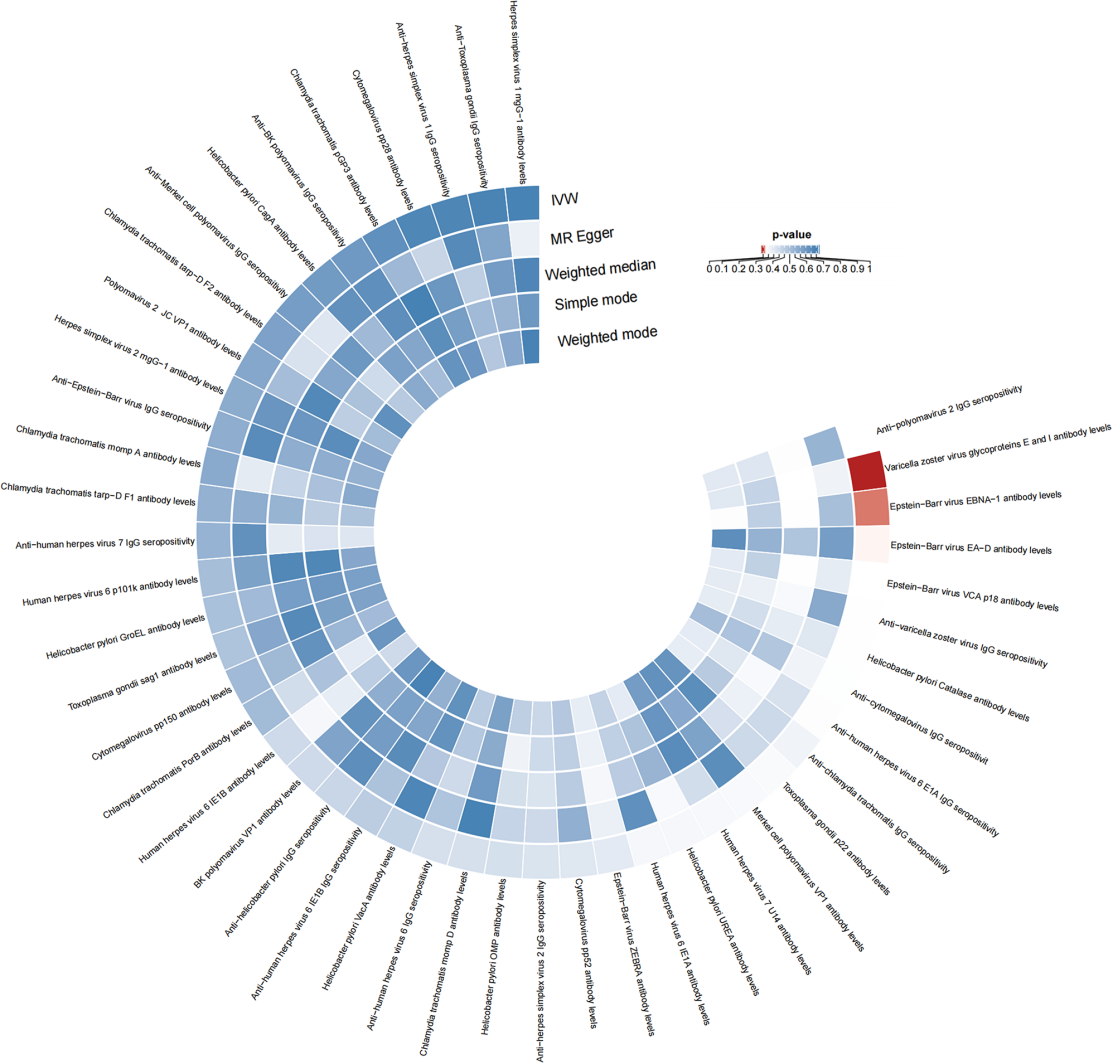
Causality of 46 antibodies on AD. The red color shows that there is statistical significance with a p-value less than 0.001. IVW, the inverse variance weighted method.The concentric circular heatmap displays the results of various analytical methods from the outermost to the innermost layer, in the following order: IVW, MR-Egger, weighted median, simple mode, and weighted mode

The causal relationships of 46 antibodies associated with antibody-mediated immune responses to infectious disease agents on AD are depicted in Fig. 2 and detailed in Tables S3. Using the IVW method, we tested the 46 antibodies and identified 2 that showed a causal association with AD. The specifics of the SNP information (SD, R^2^, F) for the 2 significant antibodies in the MR analyses are provided in Tables S1-2.

**Fig. 3:**
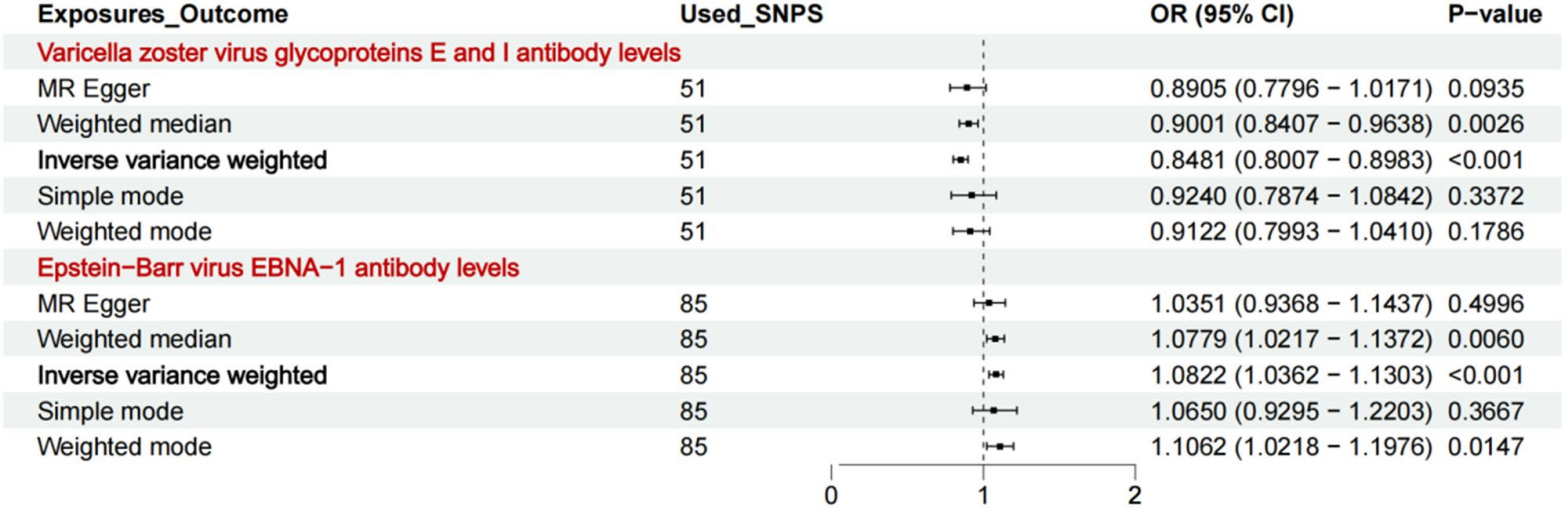
Association of antibody-mediated immune responses to infectious diseases agents with AD using Mendelian randmization

Varicella zoster virus glycoproteins E and I antibody, Epstein-Barr virus EBNA-1 antibody showed causal association with AD (Fig. 3). The risk of AD may be inversely linked to [Varicella zoster virus glycoproteins E and I antibody levels(OR = 0.8481, CI: 0.8007–0.8983, *P* < 0.001) and positively associated with Epstein-Barr virus EBNA-1 antibody levels (OR = 1.0822, CI: 1.0362–1.1303, *P* < 0.001). In summary, Varicella zoster virus glycoproteins E and I antibody functioned as protective factors; however, EBNA-1 antibody functioned as an anti-protective factor.

### Influence of AD on 46 antibodies

We also analysed the causal effect of AD on antibodies, the insufficiency of robust IVs impeded the establishment of conclusive causal inferences.

**Fig. 4:**
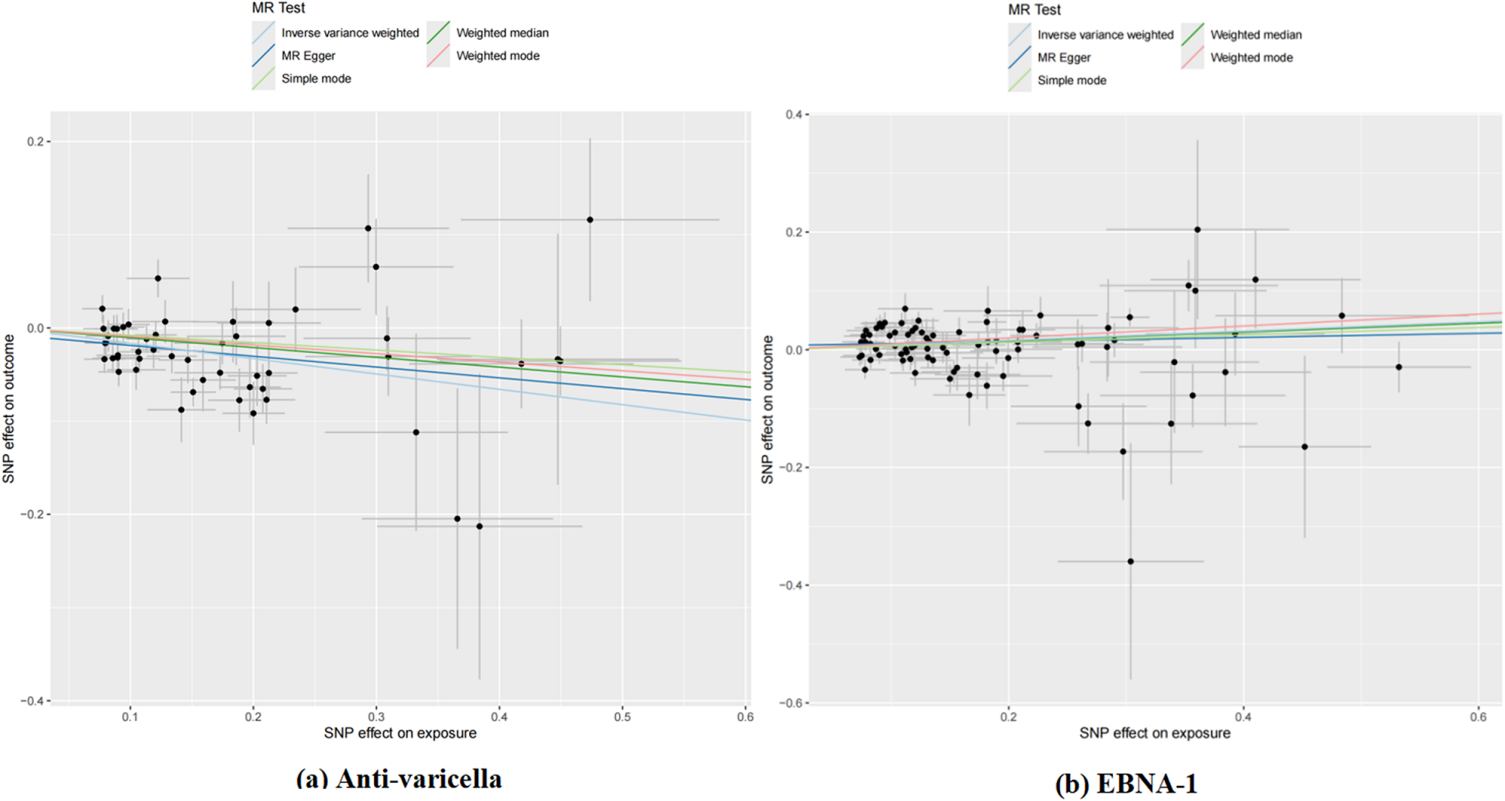
Scatter plots of each antibody associated with the risk of AD. (**a**) Anti-varicella, Anti-varicella zoster virus IgG seropositivity; (**b**) EBNA-1, Epstein-Barr virus EBNA-1 antibody levels(EBV-EBNA-1)

**Fig. 5:**
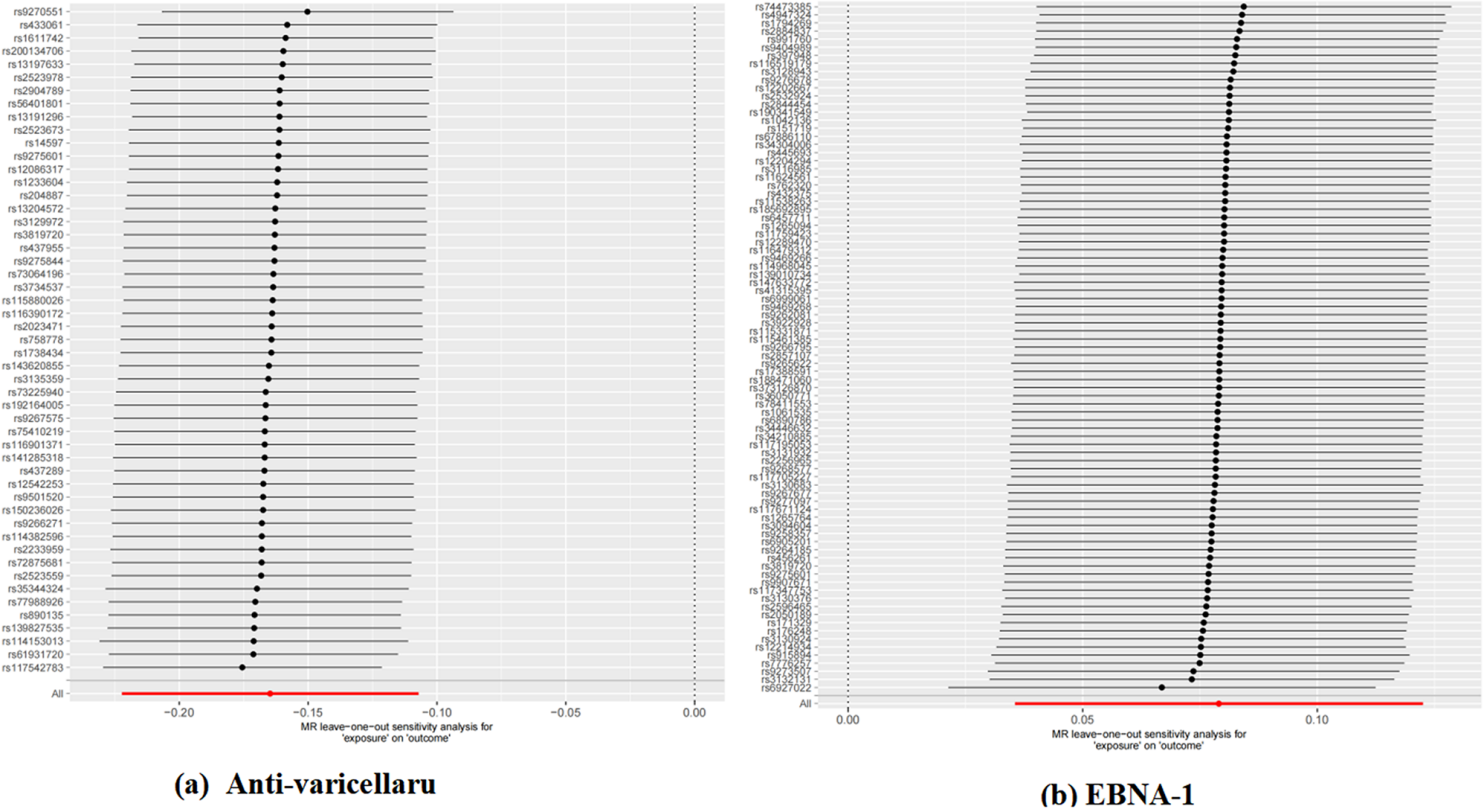
Leave-one-out analysis of each antibody associated with the risk of AD. (**a**) Anti-varicella, Anti-varicella zoster virus IgG seropositivity; (**b**) EBNA-1, Epstein-Barr virus EBNA-1 antibody levels(EBV-EBNA-1)

**Fig. 6:**
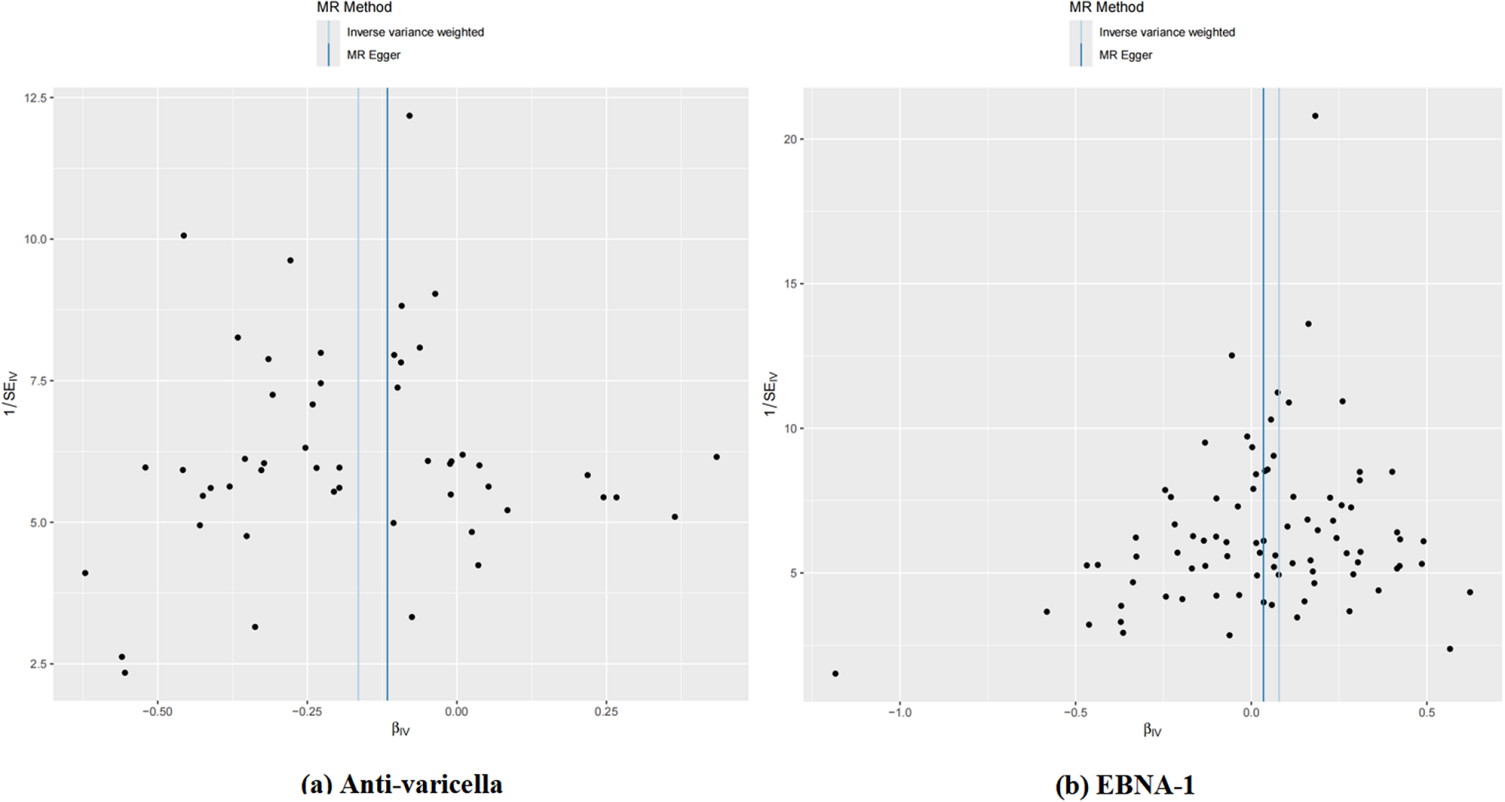
Funnel plots of each antibody associated with the risk of AD. (**a**) Anti-varicella, Anti-varicella zoster virus IgG seropositivity; (**b**) EBNA-1, Epstein-Barr virus EBNA-1 antibody levels(EBV-EBNA-1)

Heterogeneity was observed in the two antibodies. The MR Egger regression intercept did not identify any SNP pleiotropy (Table 1). The scatter plot indicates that Varicella zoster virus glycoproteins E and I antibodies may have a protective effect on AD, while Epstein-Barr virus EBNA-1 antibody levels may increase the risk of AD. MR analysis includes the IVW method, MR Egger method, weighted median method, and simple mode, all represented in scatter plots with specified weights. An upward sloping line indicates a positive correlation between infectious disease antibodies and AD, while a downward sloping line indicates a protective association (Fig. 4). No outliers were detected between infectious disease antibodies and AD using the retention method analysis (Fig. 5), indicating that the revealed causal relationship is not influenced by instrumental variables. Furthermore, the funnel plots of all results confirm the absence of horizontal pleiotropy (Fig. 6).

### Sensitivity analysis

**Table 1:** Heterogeneity and pleiotropy analysis of mendelian randomization analysis

**Exposure**		**MR-Egger intercept**		**Cochrane’s Q IVW**		**Cochrane’s Q IVW Egger**		
		**intercept value**	**P Value**	**Q Value**	**P Value**		**Q Value**	**P Value**
Varicella zoster virus glycoproteins E and I antibody levels		-0.0071	0.4291	91.9067	**<0.001**		90.7297	**<0.001**
Epstein-Barr virus EBNA-1 antibody levels(EBV-EBNA-1)		0.0071	0.3341	169.6140	**<0.001**		167.7068	**<0.001**

## Discussion

This study employed Mendelian randomization to explore potential associations between antibody-mediated immune responses to infectious disease agent and the risk of AD. Our findings indicate that varicella zoster virus glycoproteins E and I antibodies are linked to a reduced risk of AD. Conversely, elevated levels of Epstein-Barr virus EBNA-1 antibodies are associated with an increased risk of AD. These insights highlight the intricate interplay between immune responses and neurodegenerative diseases.

### Antibody-mediated immune responses to varicella zoster virus infections reduced AD risk

This study, through Mendelian randomization, found that Varicella zoster virus glycoproteins E and I antibody levels are associated with a reduced risk of AD.The presence varicella zoster virus glycoprotein E and I antibodies typically indicates that an individual has acquired immunity to varicella zoster virus, either through previous infection or vaccination [21, 22]. Varicella zoster virus infection, particularly shingles, has been linked to an increased risk of AD and other dementias [23–25]. Some studies have also found that the level of anti-herpes simplex virus IgM is negatively correlated with the level of amyloid protein in plasma [26]. The currently widely accepted view is that lower levels of amyloid protein in plasma are a possible short-term risk factor for the development of dementia, while accelerated deposition of amyloid protein in the brain occurs in parallel with a decrease in the level of amyloid protein in plasma. Therefore, this evidence also indirectly suggests that the body may accelerate the production of amyloid protein in the brain while resisting the production of IgM by the virus. Although varicella zoster virus itself does not directly cause the hallmark pathologies of AD, such as β-amyloid and tau protein accumulation, it may contribute indirectly by inducing neuroinflammation and reactivating latent herpes simplex virus type 1 in the brain, which is strongly associated with AD pathogenesis [27, 28]. Vaccination against shingles and antiviral treatments have been shown to decrease the risk of dementia, further supporting the potential involvement of varicella zoster virus in AD development [29].Therefore, controlling active herpesvirus infections may be more critical for reducing AD risk than merely increasing antibody levels [30].This finding underscores the potential role of varicella zoster virus vaccination in mitigating the risk of AD.

#### Antibody-mediated immune responses to epstein-barr virus infections increased AD risk

Epstein-Barr virus (EBV) is a widely spread herpesvirus that infects over 90% of adults worldwide for a long time [31]. EBNA-1 antibody levels can evaluate the immune status and past infection status of the body. A cohort study report suggests that the use of antiviral drugs in herpesvirus patients is associated with a reduced risk of dementia [32, 33]. Currently, the specific mechanism linking EBV infection and AD is not clear, but existing research has proposed several possible mechanisms. Continuous EBV infection and repeated reactivation may promote the formation of protein plaques in the brain [34]. The study by Carbone et al. showed that the duration of the EBV incubation period may exacerbate the systemic immune response and induce changes in the inflammatory process, leading to a decrease in cognitive ability during aging [35]. Our MR analysis found that Epstein-Barr virus EBNA-1 antibody levels increase the risk of AD, suggesting potential effects of immune mechanisms. Further research is needed to determine whether EBV reactivation is involved in triggering the onset or progression of AD.

#### Limitation

This study employs a Mendelian randomization approach to uncover the potential associations between antibody-mediated immune responses to infectious diseases agents and the risk of AD, yet it acknowledges several limitations.

Firstly, The results exhibit heterogeneity in antibody levels for Varicella zoster virus glycoproteins E and I, and Epstein-Barr virus EBNA-1. This variability may arise from population differences and co-infections, potentially affecting the accuracy and generalizability of our findings. Secondly, the data are primarily derived from the UK Biobank and FinnGen consortium, predominantly consisting of individuals of European ancestry, which may limit the generalizability of the findings. Additionally, the constraints related to the database and sample size, along with the inability to validate findings across multiple databases, impact the robustness of the study. Our study did not exclude SNPs from the HLA region, which is associated with the immune system and exhibits linkage disequilibrium, potentially influencing AD risk.

While the study offers new insights into the potential causal relationships between viral infections and AD, these results do not directly establish causality. Further epidemiological research, laboratory studies, and clinical trials are necessary to validate these findings, with large-scale, multi-ethnic population studies required to confirm and extend these observations.

## Conclusion

This study, employing Mendelian randomization, discovered that antibody-mediated immune responses to infectious diseases agents, such as anti-varicella zoster virus antibodies, are associated with a decreased risk of AD, while antibodies against Epstein-Barr virus, correlate with an increased risk. These findings highlight the complexity of immune system responses and lay the groundwork for future research to further explore the role of immune modulation in the pathogenesis of AD. Although the study provides significant insights, broader epidemiological and experimental research is necessary to validate these conclusions.

## Electronic supplementary material

Below is the link to the electronic supplementary material.

Supplementary Material 1

Supplementary Material 2

Supplementary Material 3

## Data Availability

No datasets were generated or analysed during the current study.
